# Fluoride exchange by glass-ionomer dental cements and its clinical effects: a review

**DOI:** 10.1080/26415275.2023.2244982

**Published:** 2023-08-18

**Authors:** John W. Nicholson, Sharanbir K. Sidhu, Beata Czarnecka

**Affiliations:** a Dental Materials Unit, Bart’s and the London Institute of Dentistry, Queen Mary University of London, London, UK; b Bluefield Centre for Biomaterials, London, UK; c Centre for Oral Bioengineering, Institute of Dentistry, Bart’s & The London School of Medicine and Dentistry, Queen Mary University of London, London, UK; d Department of Biomaterials and Experimental Dentistry, University of Medical Sciences, Poznań, Poland

**Keywords:** Glass-ionomer, fluoride, anti-caries

## Abstract

The topic of fluoride release and uptake by glass-ionomer (glass polyalkenoate) dental cements is reviewed. The study was based on a literature search carried out using PubMed. The main key words used were *glass-ionomer* and *fluoride*, and further refinements were made by adding the keywords *anti-microbial*, *anti-caries* and *remineralization*. Papers were selected from the initial search, which concentrated on fundamental aspects of fluoride release, including kinetics and the influence of the cement composition, and resulting clinical performance against caries. Other relevant papers were cited where they added useful and relevant data. From these published papers, it was possible to explain the detailed mechanism of fluoride release by glass-ionomer cements and also its uptake. Fluoride release has been shown to be a two-step process. In neutral solutions, the steps can be divided into early wash-out and long-term diffusion. In acid conditions, the early wash-out remains, though with greater amounts of fluoride released, and the long-term release becomes one of slow dissolution. The effect of fluoride on the viability of oral micro-organisms has been described, and glass-ionomers have been shown to release sufficient fluoride to reduce the size and viability of adjacent populations of oral bacteria. The effect of low levels of fluoride on the remineralization of tooth tissue has been considered. Levels needed to increase remineralization are much lower than those needed to adversely affect oral bacteria, from which we conclude that glass-ionomers release sufficient fluoride to promote remineralization. Despite this, there remains uncertainty about their overall contribution to sound oral health, given the widespread use of other sources of fluoride, such as toothpastes.

## KEY MESSAGES

- Glass-ionomer cements of both types (conventional and resin-modified) release fluoride for considerable periods of time after setting.
- They release sufficient fluoride to inhibit the growth of oral bacteria, and this level is more than sufficient to promote remineralization of tooth mineral.
- This shows that these materials provide sufficient fluoride to inhibit caries though, with the widespread use of fluoridated products such as toothpaste, this may have only limited benefit on the oral health of patients.

## Introduction

Glass-ionomer cements, formally called glass polyalkenoate cements, both conventional and resin-modified, have a variety of uses in dentistry [1,2]. These include dental restoratives, fissure sealants, luting cements [3] and adhesives for orthodontic brackets [4].

The original glass-ionomer cements, now often referred to as ‘conventional glass-ionomers’ were made from special basic glass powders of the types shown in Table 1. They were reacted with aqueous polyacrylic acid solution [1]. Since then, various alterations and improvements have been made. For example, (+)-tartaric acid can be used as an additive to control the setting reaction [5], acrylic-maleic acid copolymer can be used instead of polyacrylic acid as the polymer component [6], and the polymer can be added in dried form to the glass powder [7], which effectively increases the concentration of the polymer in the cement and improves the mechanical properties of the set material [8].

**Table 1.** Pre-firing compositions of G200 and G338 [1, 13].

Component	G200 composition/%	G338 composition/%
SiO_2_	30.1	24.9
Al_2_O_3_	29.9	14.2
AlF_3_	2.6	4.6
CaF_2_	34.5	12.8
NaF	3.7	–
AlPO_4_	10.0	24.2
NaAlF_6_	–	19.2

The most substantial modification has been the addition of a water-soluble monomer, 2-hydroxyethyl methacrylate, together with an appropriate polymerization initiator, to create resin-modified glass-ionomers [9]. In most products, this initiator is light-activated, so that resin-modified glass-ionomers are typically cured, in part at least, photochemically [9,10]. Resin-modified glass-ionomers also undergo setting via reaction of the polymeric acid with the basic glass powder, and this means there are two cure processes in these materials. The resulting material has a complex microstructure, the details of which depend on how quickly the light-cure polymerization is initiated after mixing the cement [11].

Both types of glass-ionomer are able to release fluoride once set [1,2]. Fluoride is present as a component of the glass. This is because the ionomer glass is made from a mixture that includes either calcite (CaF_2_) or cryolite (Na_3_AlF_6_) (see Table 1). These substances are present in the pre-firing glass mixture in order to lower the fusion temperature of the glass [12]. Glasses containing either of these substances can be melted at temperatures of around 1200 °C rather than in the region 1550–1600 °C, which would be necessary if they were not present [13]. Table 1 shows the pre-firing compositions of two significant ionomer glasses, G200, the first technically successful glass formulation, and G338, a widely studied formation that is close to most currently used ionomer glasses.

As well as reducing the fusion temperature of the glass, the presence of fluoride is found to improve the compressive strength of the set cement [13]. Fluoride-containing glasses typically give rise to set cements with compressive strengths of at least 200 MPa, whereas fluoride-free glasses have values around 100 MPa or lower [13,14].

A further advantage of including fluoride in the glasses is that it reduces their refractive index, and thereby improves the appearance of the set cement [15]. This phenomenon was not considered explicitly in the initial development of ionomer glasses, but emerged in much later research. However, the early empirical research was guided by the need to fabricate translucent glasses that were able to form cements with optical properties that resembled the natural tooth to some extent [16]. To an extent, this guided the level of fluoride included.

Despite the inclusion of fluoride, and the resulting ability of glass-ionomer cements to release it in a sustained way for long time periods, the clinical effectiveness of this release is unclear. Some authors claim that it is beneficial, others that it has no effect, probably because such small amounts are released. While studies continue on the detailed chemistry of fluoride release, and also uptake, the question of the clinical benefit of fluoride release remains largely unanswered. This review aims to address this question, together with giving an account of our current understanding of fluoride release and uptake.

## Methods

The study involved a search carried out with PubMed using the key words *glass-ionomer* and *fluoride*, with further refinements through adding the following keywords in turn: *anti-microbial*, *anti-caries* and *remineralization*. The initial search identified 1393 references, and each of the refinements produced many fewer references, and only *remineralization* identified more than five papers. The main selection from the initial list of papers concentrated on studies that described: (i) fundamental aspects of fluoride release, including kinetics and the influence of the cement composition and (ii) overall clinical performance, including the effectiveness of the fluoride against caries. In addition, other relevant papers previously known to the authors have been cited where they provide useful and relevant data. Using all this published information, the detailed mechanism of fluoride release by glass-ionomers and its uptake can be explained. In addition, its effect on both oral micro-organisms and demineralized tooth tissue can be understood, and a reasonable judgment can be made as to the clinical effectiveness of the fluoride release.

### Fluoride release from glass-ionomer cements

Both types of glass-ionomer cement are capable of releasing fluoride and, despite occasional claims that one or the other type releases more fluoride, they are generally reported as releasing comparable amounts [17,18]. Certainly, there is no fundamental reason why either conventional or resin-modified glass-ionomers should release more.

Initially, release of fluoride was assumed to occur as part of an erosion process [19]. However, it soon became clear that fluoride could be released without any obvious erosion occurring to the cement [20], and this has been confirmed in all subsequent studies [21]. Quite early on, the occurrence of at least two release processes was observed [22], one of which involved diffusion [23]. The current view of fluoride release is that it comprises a relatively large amount being released in the first few days, or possible weeks, followed by a steady low-level of release [24,25]. The first step is known as ‘early wash out’ and is hardly affected at all by the surroundings. The second step, by contrast, varies with the pH of the surroundings. Around neutral pH, it is a diffusion process, i.e. one that shows a dependence on the square root of time, √*t*. In acidic pH conditions, it becomes a slow erosion process, depending directly on time. Kinetic equations describing these two release profiles have been determined [24,25] and are:

$${\left[F\right]}_{c}=\;{\left[F\right]}_{1}t/\left(t\;+\;t_{½}\right)\;+\;\beta \surd t\;\left(\text{neutral pH conditions}\right)$$

$${\left[F\right]}_{c}=\;{\left[F\right]}_{1}t/\left(t\;+\;t_{½}\right)\;+\;\alpha t\;\;\left(\text{acidic pH conditions}\right)$$

In these equations, [*F*]_1_ is the amount of fluoride released by early wash out, and can be determined from a plot of fluoride release against square root of time in neutral conditions. Such a plot shows a slope of *β* and an intercept of approximately [*F*]_1_. The *t*_1/2_ term can be calculated once the [*F*]_1_ term is known. In this way, all of the terms in each equation can be found, and the quality of fit for the calculated value of [*F*]_c_ can be compared with the experimental values. Studies have shown that there is excellent agreement between calculated and experimental values [24,25].

The total amount of fluoride released also increases when the external conditions are acidic rather than neutral [26–28]. This has been confirmed in numerous studies [24] and appears to be associated with the change in long-term release kinetics.

The chemistry of the fluoride release process has been studied, and the most widely published view is that it occurs by ion exchange, with fluoride ions released from the cement being replaced by hydroxide ions from the storage solution [29–31]. An exchange of this type would explain why fluoride release is not associated with any observable loss of structure by the cement. An important feature of this suggested mechanism is that the removal of hydroxide ions from the aqueous solution would increase the relative concentration of hydrogen ions, H^+^, thereby resulting in a reduction in the pH [30]. If this pH change is not checked, it could fall to below 4, a value at which the hydroxyapatite tooth mineral would dissolve and re-precipitate as calcium fluoride. Even at a slightly higher pH, i.e. up to 4.5, hydroxyapatite would dissolve, but the substance to precipitate would be fluorapatite rather than CaF_2_ [30].

Unfortunately, this simple mechanism is not supported by the evidence. Studies of pH change of storage solutions for glass-ionomer cements show that, rather than the pH being reduced over time, it is actually increased. In other words, if anything, cements remove H^+^ ions from solution, not hydroxide ions [32]. For example, one study of conventional glass-ionomer cement stored in water that was initially at pH 5.9 found the pH to shift between 6.7 and 6.9 [30]. This change was attributed to the relatively large amounts of unreacted basic glass powder in the set cement. The possibility of an initial hydroxide-for-fluoride exchange process is not ruled out completely by these findings, but the observed shift in pH shows that it can only be a minor part of the release process.

A number of other ions have been found to be liberated along with fluoride when glass-ionomers are stored in aqueous media. These include sodium, aluminum, silicate and phosphate. As with fluoride, higher amounts of each species are eluted in acidic conditions than in neutral ones [27,32]. In addition, either calcium or strontium is also released from cements in acidic solutions, a feature that is attributed to attack on the strongly basic regions of the glass filler rich in CaO (or SrO).

Another feature, which has been reported in the literature but is largely ignored, is that in acidic conditions, more of the fluoride is released as complexes rather than as free F^–^ ions [27,32]. The two types of fluoride may be distinguished using a fluoride-selective electrode [32]. This device is able to determine F^–^ ions only, and fluoride in any other form, for example combined into a complex ion, is not detected. The overwhelming majority of studies forcibly decomplex all of the fluoride present by adding total ionic solubility acid buffer (TISAB) solution prior to measuring fluoride in the storage solutions.

A minority of studies, by contrast, have measured fluoride content of storage solutions without any TISAB present, and compared the results with the measurements on the same solutions following the addition of TISAB. This has allowed the researchers to determine the relative amounts of free and complexed fluoride, and to demonstrate that acidic storage conditions generally cause fluoride to be released as complexes. Data from one such study are shown in Table 2 using storage solutions from the glass-ionomer brand Fuji IX GP. Results show that in lactic acid solution (pH 2.7), all of the fluoride released was complexed, whereas in water (pH 5.9) only just over half was complexed, and the rest was free.

**Table 2.** Elements released from Fuji IX GP into water and lactic acid solution (standard deviations in parentheses) [32].

	Water	Lactic acid solution
pH	7	4
Free fluoride/ppm	7.4 (2.5)	0.0 (0.0)
Total fluoride/ppm	16.9 (2.5)	28.9 (6.5)
Complexed fluoride/%	56.2 (8.3)	100.0 (0.0)
*Solution composition*		
Si	4.05 (0.04)	21.32 (0.24)
P	0.58 (0.09)	3.53 (0.05)
Al	5.01 (0.05)	35.03 (0.41)
Sr	4.74 (0.09)	52.43 (0.35)

The nature of the complex(es) has not been determined beyond all doubt. In principle, fluoride can complex with either protons or aluminum ions. With protons, undissociated hydrofluoric acid, HF, is the major product [33] whereas with aluminum, a variety of complex ions can be formed [34], such as AlF_4_^–^. Evidence from solution studies suggests that the pH at which protons form stable complexes with F^–^ ions is low, i.e. below 4–4.5 [33] from which it may be inferred that aluminum complexes are the species that form.

Interestingly, whatever the nature of the complexes, they are able to fluoridate hydroxyapatite, as shown in Table 3 [32]. Those based on aluminum have been found experimentally to mainly dissociate as they interact with hydroxyapatite. These complexes were found to dissociate in contact with hydroxyapatite powders, leaving fluoride in the powder but only traces of aluminum [32]. As results in Table 3 also show, complexed fluoride is taken up more readily by hydroxyapatite than un-complexed fluoride, a surprising finding that is difficult to explain.

**Table 3.** Change in total fluoride concentration (ppm) with time for 5 cm^3^ extract solution in contact with 0.100 g synthetic hydroxyapatite powder (experiments in triplicate; standard deviations in parentheses) [32].

Exposure time/min	Water	Lactic acid solution
0	16.9 (2.5)	28.9 (6.5)
5	6.9 (1.4)	0.0 (0.0)
30	1.6 (0.7)	0.0 (0.0)

With the multi-component glasses used in clinical grades of glass-ionomer cement, there is the possibility that some of the fluoride is released by a simple dissolution process, involving NaF dissolving out of the cement. Certainly, these cements give substantial sodium release [27], and studies have shown that the kinetics of this release matches that of fluoride [35]. Against that, some of the experimental glasses studied have had much simpler compositions, and not contained any sodium, or any other ion that can form simple soluble salts with fluoride [31]. In these cements, therefore, it may be that fluoride release occurs mainly by a hydroxide-for-fluoride exchange. In the absence of any studies on the pH changes with these cements, this possibility remains speculative.

### Fluoride exchange

Fluoride has been shown to be capable of being taken up by glass-ionomer cements, and there have been numerous publications describing this phenomenon [36–39]. Unfortunately, these studies typically have not studied the whole process, but only considered the increase in fluoride release following exposure to a solution containing aqueous fluoride ions. This has led to the suggestion that fluoride can be readily exchanged by glass-ionomers, depending on the external fluoride concentration [40]. There has also been a claim that this makes them especially suitable in regions of high caries challenge [40]. Neither statement is true. A few key studies have demonstrated that most of the fluoride taken up is retained by the cement [41,42], and a recent study using ^19^F NMR spectroscopy showed that it forms an insoluble species with aluminum within the cement [42]. This aluminum–fluoride complex is too insoluble to be readily re-released. Although exposing a cement to a fluoride solution causes a rise in the total fluoride released, only a small proportion of the fluoride taken up is released again.

This possible fluoride exchange means that experimental fluoride-free cements can release fluoride once they have been exposed to a solution of fluoride ions [43]. The importance of this property is open to question because, in the mouth, exposing a cement to fluoride solutions would also expose the adjacent tooth to fluoride. Under these circumstances, fluoride would be taken up by the hydroxyapatite mineral phase of the tooth. And any subsequent fluoride release by the glass-ionomer could have only a minimal effect, if any. Consequently, any fluoride exchange by glass-ionomers can have only very limited therapeutic benefit.

The ability to exchange fluoride with the surroundings, though limited, is a property of glass-ionomers of both types but is not shown by composite resins [44]. Composites can be made to release fluoride, for example, by adding compounds such as ytterbium fluoride, but these materials cannot take up fluoride when exposed to fluoride solutions. With composites, once fluoride has been released, there is no capacity for recharge.

A somewhat neglected study reported that zinc phosphate dental cement can also take up fluoride from solution and re-release it into deionized water [45]. This seems to be due to the presence of the phosphate groups in the cement. However, dental zinc phosphate cement contains substantial amounts of aluminum, added to control the rate of the setting reaction. This aluminum could also interact with fluoride in the way shown recently by glass-ionomer cements [42]. In both types of cement, aluminum may be bound to phosphate, but also have some coordination sites available to which fluoride ions can bond. Whatever the mechanism, these results for zinc phosphate cement show that the ability to take up and re-release fluoride is not restricted to glass-ionomers [45].

Lastly, we note that fluoride can be taken up by glass-ionomer cements from several different sources. Several experimental studies have used solutions of either sodium or potassium fluoride, typically at concentrations of 1000 ppm in fluoride [46,47]. Other experiments have shown that fluoride can be delivered by toothpaste [48], mouthwashes [49,50] and topically applied fluoride gels [51]. The fact that fluoride is found in these formulations in association with various counterions suggests that the formulation of the fluoridating medium is relatively unimportant, and that the affinity of glass-ionomers for fluoride is enough to overcome any interaction of fluoride ions with other components of these mixtures.

### Measurement of fluoride released

One of the features of studies of fluoride release is that there are no standard conditions or units of measurement. Cement samples have been of various shapes. The most frequently used are discs, though these vary from relatively small, i.e. 5 mm diameter × 2 mm thick [52], through medium, i.e. 11 mm diameter × 1.5 mm thick [53] to relatively large, i.e. 20 mm diameter × 1 mm thick [54,55]. Other studies have used cylindrical samples [46,56] or powdered cement [32]. Volumes of storage solution have varied, with 5 cm^3^ being common [57] but not universal. Lastly, amounts released have been quoted in several different units, with μg/cm^2^ being the most common, but with ppm being also quite frequent.

This wide variety in experimental details makes it impossible to compare results from different studies. The fact that glass-ionomers of both types release fluoride is well established, but how the different brands compare with each other can be difficult to determine, as is the way results on experimental samples relate to the geometry of restorations used clinically.

The units of release of μg/cm^2^ have the advantage that they recognize that fluoride leaves the cement across the interface with the surroundings. However, they do not give any idea of the concentration of fluoride that results in the saliva immediate adjacent to the cement. This is a problem, because it is the concentration that matters in influencing the balance between demineralization and remineralization of the teeth.

### Fluoride and dental caries

The mineral phase of the tooth consists mainly of hydroxyapatite, Ca_10_(PO_4_)_6_(OH)_2_ [58]. This substance is very sparingly soluble under neutral conditions, with an estimated solubility product, *K*_sp_, in the range 10^−57^ to 10^−60^ [59]. However, under mildly acidic conditions, e.g. pH around 5, it undergoes dissolution, and is the basis of clinically observed active caries [60,61]. This is known as demineralization and occurs *in vivo* as a result of the action of biofilm, which colonizes the dental hard tissues [62], and then metabolizes reducing sugars to produce a cocktail of weak organic acids, of which the most abundant is lactic acid (88.2 ± 8.3%) [60,63]. These acids then dissolve the mineral phase of the tooth, which begins the process of caries. Left unchecked, this can have a variety of clinical consequences, leading to significant loss of the mineral phase of the tooth and the formation of a carious lesion. Further progress of the condition can lead to systemic infection, with bacteria penetrating to the dental pulp and entering the patient’s bloodstream.

Under the conditions in a healthy mouth, the ion content and buffering capacity of the saliva can repair the damage done by demineralization in a process known as remineralization. This means that there is a balance between these two processes [64]. This balance is frequently described, incorrectly, as an equilibrium [65], but it is in fact a steady state. Left to its own devices, the balance will ensure the continued precipitation of hydroxyapatite mineral to replace that lost by dissolution, and maintain the tooth in place. Dental caries occurs when conditions favor demineralization, typically when the biofilm is provided with ample carbohydrates, and is able to produce its mixture of organic acids [61].

The demineralization that occurs is reversible and remineralization can be encouraged by shifting the pH of the microenvironment to values above 5.9 [63] and preferably closer to 7.0 [65]. The calcium and phosphate ions needed for remineralization are present in the saliva, and they precipitate onto the existing mineral surfaces as an amorphous mineral layer. When the conditions are right, this layer will act as a precursor for a more organized mineral structure. The result is so-called epitaxial growth, leading to the remineralization of the damaged tissues [66].

For many years, fluoride has been known to be beneficial in inhibiting the demineralization process [67–69]. The fluoride ion operates by two mechanisms, namely (i) directly inhibiting the metabolism of microbial cells in the biofilm and (ii) enhancing the rate of the remineralization process, thereby adjusting the balance in favor of remineralization.

The biochemical effects of fluoride in the cells of micro-organisms are complicated. They appear to center around the formation of HF within the cells, a process which inhibits bacterial physiology, and leads to reduced viability [70]. Carbohydrate metabolism is inhibited [71] and this in turn leads to reduced numbers of *Streptococcus mutans* present in the oral biofilm [70]. The net effect of these changes is that there are fewer micro-organisms actively metabolizing sugars to produce lactic acid and the other organic acids that cause caries. Consequently, caries development is inhibited.

Fluoride also affects the physical chemistry of the dissolution process of hydroxyapatite. The rate at which fluoridated apatite dissolves is much lower than that of hydroxyapatite [72], and there is evidence that a very thin layer of the fluoridated substance forms on the surface of hydroxyapatite exposed to fluoride solutions [73,74]. There is also evidence that this fluoridated layer is less soluble in aqueous media than hydroxyapatite [72–75]. The presence of fluoride in the surrounding solution also reduces the rate of dissolution of hydroxyapatite [76] and promotes the remineralization process at the hydroxyapatite surface [77] thereby becoming incorporated into the newly deposited mineral phase [68]. This clearly alters the solubility of this phase, though changing the solubility does not appear to be fluoride’s main role. In addition to its effects on the solid component of the process, fluoride also reduces the solvating ability of saliva through the formation of strong hydrogen bonds with the water [68]. The overall effect is that the development of carious lesions in the enamel is arrested [78].

The concentration of fluoride needed to make a difference to the dissolution of hydroxyapatite is very low. Effects have been reported at levels down to 0.009 pm [79], although this has not been confirmed and other authors have found that such a low level of fluoride did not cause any reduction in demineralization [80]. By contrast, as results in Table 4 show, the lowest level at which an effect could be detected in the latter study was 0.015 ppm.

**Table 4.** Reduction in demineralization with resting fluoride concentration in saliva (from [80]).

Resting fluoride concentration/ppm	Reduction in demineralization/%
0.009	0
0.015	20
0.025	30
0.190	50
1.90	60

Other studies have confirmed that fluoride at a level of 0.014 ppm is capable of reducing demineralization [80] and that levels in the range 0.01–0.2 ppm induce apatite growth [81–84]. Several of these studies have used toothpastes as the source of fluoride. Although these are typically formulated with reasonable levels of fluoride, typically around 1000 ppm [85], levels of fluoride in saliva drop rapidly after brushing, so that after 30 min the concentration in saliva is around 1 ppm [86]. This level drops even further to around 0.02 ppm 12–18 h after brushing [80] but is still sufficient to shift the demineralization–remineralization balance back toward remineralization. The observed reductions in demineralization must be due to the effect on solubility and dissolution of tooth mineral, as levels of fluoride around 0.02 ppm are not sufficient to affect the growth of cariogenic bacteria [80].

### Clinical benefit of fluoride release from glass-ionomers

Many authors mention in passing that the fluoride release by glass-ionomers of both types is beneficial. Unfortunately, this is not supported by the literature, and the general opinion is that the anti-caries effect of fluoride release by glass-ionomers has not been demonstrated beyond all doubt [87]. In this section, we consider the evidence and draw some conclusions about how likely such an effect is.

Glass-ionomers were developed from the now obsolete dental silicate cement [16]. The glasses are similar in both cements, but the acid in dental silicate was phosphoric acid, which is much stronger than poly(acrylic acid). Consequently, the glass in dental silicates was correspondingly less basic. Indeed, much of the early work in the development of glass-ionomers was aimed at increasing the basicity of the glass powder, so that it could reach the point where the cement set quickly enough to be practical in clinical situations, i.e. was well hardened within 2–3 min [16].

A feature of dental silicate cements was that they, too, released fluoride when set [88,89]. This behavior was never studied in great detail and, like glass-ionomers, arose accidentally because fluoride compounds were included in the pre-firing glass mixture to lower the fusion temperature of the glass. As a result of this fluoride release, it was known from the 1940s that dental silicates had anti-caries properties [88].

The initial doubts about the clinical effectiveness of glass-ionomers against caries emerged from work by Mjör [90] and Mjör and coworkers [91]. They carried out a series of studies that were practice-based and collected the opinions of groups of general dental practitioners. These practitioners reported that they could find no difference between the anti-caries properties of glass-ionomers and other restorative materials, and that secondary caries was just as prevalent with them as with any other material. Shortly afterwards, Randall and Wilson published a review [92] which questioned the validity of published studies that found glass-ionomers to be effective against caries. According to Randall and Wilson, such studies typically used small cohorts of patients, and this reduced the power of their studies and raised doubts about the validity of their findings [92].

In fact, the studies of Mjör et al. are at least similarly questionable, relying as they did on unsubstantiated opinions of busy dental practitioners, and without presenting any evidence to show that their records were either reliable or accurate. But Mjör’s work succeeded in raising concerns about the anti-caries effectiveness of glass-ionomers, and these continue to the present day [87].

Despite the doubts, there is evidence of effectiveness in some clinical applications, at least. Studies have shown that resin-modified glass-ionomers are good adhesives for orthodontic brackets [93,94] and, in particular, the problem of white spot lesions adjacent to cemented brackets that arise from enamel demineralization with certain adhesives, do not occur with glass-ionomers. Studies have also shown that resin-modified glass-ionomers as bonding agents for orthodontic brackets are associated with reduced enamel demineralization [95].

Glass-ionomer sealants have also been shown to be effective against caries [96] and enamel adjacent to glass-ionomer fillings has been shown to be harder [97] and better able to resist attack by acids than enamel at a distance from such fillings [97–99]. There are some other indications of clinical effectiveness, notably that remineralization has been found to occur around glass-ionomer fillings in both enamel and dentine and with conventional and resin-modified glass-ionomers [100–103]. In all cases, this seems likely to arise from the action of fluoride released by the cements. The anti-caries effect was confirmed in a detailed review of all the published evidence at the time the paper was published [104] though the same authors concluded that evidence of remineralization was limited.

As we have seen, one of the effects of fluoride is to inhibit the metabolism of *Streptococcus mutans* bacteria in the biofilm. The question is, how much fluoride does it take for this effect to occur? A further question is, do glass-ionomers release enough fluoride to cause this inhibition? The answer turns out to be complicated.

A feature of *S. mutans* is that it can withstand regular cycles in pH from 7 to 3 or lower, and back again. This cycle occurs naturally in the mouth, due to the metabolism of carbohydrates consumed by the host. This leads to the formation of weak organic acids, and a reduction in pH [105,106], followed by gradual buffering by the saliva and a return to pH 7. The ability to withstand this wide range of pH values is known as acidurance, and it is affected by the presence of fluoride. Moreover, the amount of fluoride that is needed to affect the metabolism of *S. mutans* varies with pH and under acidic conditions, the amount is lower than at pH 7 [107].

The amount of fluoride needed to affect *S. mutans* populations in the mouth has been determined. Survival rates dropped dramatically at concentrations of 500 mmol dm^−3^ [108] and levels as low as 10 mmol dm^−3^ were sufficient to inhibit metabolism and render the bacteria unable to metabolize carbohydrate [69,109]. These values are equivalent to approximately 10,000 and 200 ppm, respectively.

Much lower concentrations have been shown to affect the viability of the main cariogenic bacterium, *Streptococcus mutans*, *in vivo*. Growth has been shown to be inhibited and metabolism to be affected, so that acid production is reduced. In many cases, total numbers of organisms in the population drop substantially [110–118]. Glass-ionomer restorations have been shown to raise levels of fluoride in the saliva of children to levels that affected *S. mutans* and reduced the numbers of these micro-organisms able to actively metabolize carbohydrates [119,120]. Concentrations of fluoride that caused this effect were measured using various brands of glass-ionomer cement and, after 3 weeks, levels were between 0.8 and 1.2 ppm. These fell to 0.3–0.4 ppm after 6 weeks, where they remained after a year [119,120].

The concentrations of fluoride in both saliva and also in the plaque increase when glass-ionomer cements are present in the mouth [100]. The levels involved, which cause adverse effects on *S. mutans*, are much higher than those needed to shift the balance toward remineralization. One review has suggested that a concentration in the range of 0.03–0.08 ppm is needed to promote remineralization, though, as we have seen, this effect can actually be achieved *in vitro* at levels as low as 0.015 ppm [80].

Data from the studies of the effect of fluoride from glass-ionomers on *S. mutans* are clear: sufficient fluoride is released to cause measurable damage to biofilms containing mainly *S. mutans*. Amounts needed for these adverse effects are around 10–20 times those needed to promote remineralization. Hence, it is obvious that the amount of fluoride released by glass-ionomers must be enough to promote remineralization *in vivo*.

The question of whether this is beneficial clinically remains, however. This is because of the widespread use of fluoride, for example in drinking water and in toothpastes throughout the world [87,121]. These sources are capable to providing the levels of long-term fluoride necessary to promote remineralization and inhibit caries by themselves. Any additional fluoride from glass-ionomers is unlikely to add to the beneficial effects arising from the use of fluoridated toothpastes. This may be the reason that no difference in the incidence of caries adjacent to restorations has been found between composite resins and glass-ionomer cements [122].

## Conclusions

The fluoride released by glass-ionomer cements of both types (conventional and resin-modified) is sufficient to have detectable adverse effects on the oral bacteria responsible for dental caries. Several published studies suggest that it is not possible to determine whether this amount of fluoride has any clinical benefit on the demineralization-remineralization balance. However, this is not true. The amounts needed to alter this balance have been determined in *in vitro* studies, and found to be an order of magnitude less than the amount necessary to damage the oral bacteria. This means that glass-ionomers do release sufficient fluoride to have a positive effect on the remineralization of teeth. In turn, this shows that glass-ionomers are able to play a part in counteracting caries. In practice, though, this may be of only limited clinical benefit, because of the widespread availability of other sources of fluoride, notably drinking water and toothpastes.
